# 代谢组学在肺癌研究中的应用及其研究进展

**DOI:** 10.3779/j.issn.1009-3419.2025.106.20

**Published:** 2025-07-20

**Authors:** Daoyun WANG, Zhicheng HUANG, Bowen LI, Yadong WANG, Zhina WANG, Nan ZHANG, Zewen WEI, Naixin LIANG, Shanqing LI

**Affiliations:** ^1^100730 北京，中国医学科学院，北京协和医学院，北京协和医院胸外科; ^1^Department of Thoracic Surgery, Peking Union Medical College Hospital, Chinese Academy of Medical Sciences and Peking Union Medical College, Beijing 100730, China; ^2^100028 北京，应急总医院呼吸及危重症医学科; ^2^Department of Pulmonary and Critical Care Medicine, Emergency General Hospital, Beijing 100028, China; ^3^100081 北京，北京理工大学医学技术学院生物医学工程系; ^3^Department of Biomedical Engineering, School of Medical Technology, Beijing Institute of Technology, Beijing 100081, China

**Keywords:** 肺肿瘤, 代谢组学, 代谢重编程, 耐药性, Lung neoplasms, Metabolomics, Metabolic reprogramming, Drug resistance

## Abstract

肺癌，尤其是非小细胞肺癌（non-small cell lung cancer, NSCLC），是全球癌症相关死亡的主要原因。近年来，代谢组学作为一种系统生物学技术，专注于对细胞、组织或机体内小分子代谢物的全面解析，为肺癌的早期诊断、代谢特征识别和耐药机制研究提供了新的策略和方法。肿瘤的代谢重编程是肺癌发生和发展的重要驱动因素。代谢组学研究揭示，肺癌细胞可通过调控能量代谢、脂质代谢和氨基酸代谢等关键途径，适应快速增殖和侵袭转移的需求。本文综述了代谢组学在肺癌研究中的最新进展，重点探讨肺癌代谢重编程的特征、潜在代谢生物标志物的挖掘，以及代谢组学在肺癌早期诊断和耐药机制解析中的应用前景。

## Abstract (en)

Lung cancer, particularly non-small cell lung cancer (NSCLC), is a leading cause of cancer-related mortality worldwide. In recent years, metabolomics has emerged as a key systems biology approach for analyzing small-molecule metabolites in cells, tissues and organisms. It provides new strategies for early diagnosis and metabolic profiling. Additionally, metabolomics plays a crucial role in studying resistance mechanisms in lung cancer. Tumor cell metabolic reprogramming is a key driving factor in the initiation and progression of lung cancer. Metabolomics studies have revealed how lung cancer cells regulate critical pathways such as energy metabolism, lipid metabolism, and amino acid metabolism to adapt to the demands of rapid proliferation and invasive metastasis. This review summarizes the latest advances in metabolomics research in lung cancer, focusing on the characteristics of metabolic reprogramming, the identification of potential metabolic biomarkers, and the prospects of metabolomics in early diagnosis and the elucidation of resistance mechanisms in lung cancer.

肺癌是全球范围内发病率和死亡率最高的癌症，其中非小细胞肺癌（non-small cell lung cancer, NSCLC）占所有肺癌病例的85%^[1]^。尽管肺癌的治疗进入了精准医学领域，靶向治疗和免疫治疗的不断创新使部分患者的生存期有所延长，但肺癌的总体生存率仍较低，尤其是对于晚期患者而言^[2]^。现有疗法普遍面临耐药性挑战，在肿瘤异质性和耐药机制的复杂性面前，如何揭示NSCLC的耐药机制已经成为临床上亟需解决的难题。

代谢组学是一门对生物体内的代谢物进行全面、系统分析的学科。通过高通量技术如气相色谱-质谱联用（gas chromatography mass spectrometry, GC-MS）、液相色谱-质谱联用（liquid chromatography mass spectrometry, LC-MS）和核磁共振（nuclear magnetic resonance, NMR）等，代谢组学能够捕捉到细胞在不同生理和病理状态下的代谢变化^[3]^。与基因组学、转录组学不同，代谢组学能够直接反映细胞的生理状态，为肿瘤研究提供了新的维度。

近年来，代谢组学作为研究肺癌发生发展机制的重要手段，展现出在疾病早筛、分型及耐药机制解析中的广泛应用前景。在早期阶段，代谢组学可挖掘特异性代谢标志物，提升肺癌筛查的敏感性与特异性，克服低剂量螺旋计算机断层扫描（low-dose computed tomography, LDCT）假阳性率较高的局限。血清和尿液等体液代谢组学的应用也为无创诊断提供了新思路。在疾病进展过程中，代谢组学可以揭示细胞内和肿瘤微环境中糖、脂质与氨基酸代谢的重编程现象，进一步阐明其与免疫调控、转移能力之间的关联。在晚期耐药阶段，代谢适应被认为是肺癌细胞产生治疗抵抗的关键机制。耐药性是由多条通路协同改变所形成的网络效应所致，包括能量生成、抗氧化、营养物质摄取与利用等关键环节的调控。肺癌细胞通过系统性地调整多种代谢程序，实现对化疗、靶向治疗等干预手段的适应和逃逸。因此，整合多组学数据以全面理解代谢网络的变化，已成为深入认识肺癌耐药机制的重要策略。代谢组学在肺癌研究中的应用覆盖疾病发生、发展和耐药的全过程，提供了一种基于代谢变化的综合性视角，有助于推动肺癌的精准诊疗和个体化治疗策略的发展。本文旨在综述代谢组学在肺癌研究中的最新进展，重点探讨肺癌代谢重编程的特征、潜在代谢生物标志物的挖掘，以及代谢组学在肺癌早期诊断和耐药机制解析中的应用前景。

## 1 肺癌的代谢重编程

代谢重编程是指肿瘤细胞在不同行为和微环境压力下，通过改变能量代谢、氨基酸代谢、脂质代谢等多种代谢途径，支持其生长、扩散、存活以及免疫逃逸的过程^[4]^。肺癌细胞的代谢特征与正常肺泡上皮细胞有很大差异，基于肺癌组织的代谢组学研究^[5]^揭示了多个关键的代谢途径和标志物，代谢重编程在肺癌中的主要表现见图1。

**图1:**
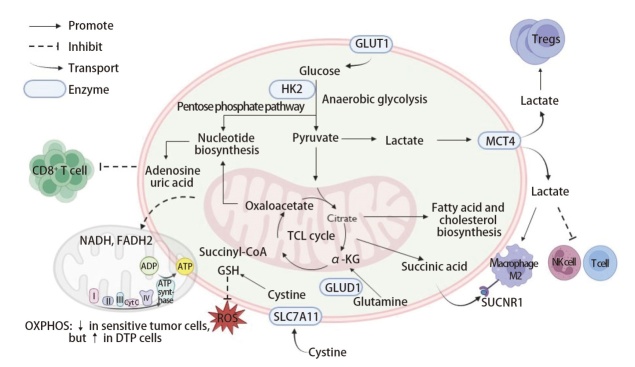
肺癌细胞的代谢重编程

### 1.1 糖和能量代谢重编程

无氧糖酵解是肿瘤细胞在有氧条件下依然优先进行的能量代谢过程，这一现象被称为Warburg效应。多项研究^[6-9]^显示，无氧糖酵解的中间产物丙酮酸和最终产物乳酸在肺癌组织中显著上调，且糖酵解旁路的中间产物果糖、山梨糖醇和赤藓酸盐等也在肺癌组织中显著增加。肺癌细胞通过无氧糖酵解快速获得能量，并通过糖异生等旁路为其合成代谢提供原料。此外，乳酸的积累不仅为肿瘤细胞提供能量和酸化微环境支持，促进肿瘤细胞的增殖和转移，还通过调控免疫细胞和免疫微环境（tumor immune microenvironment, TIME），促进免疫逃逸和肿瘤的侵袭性^[10]^。

能量代谢方面，有关研究^[7,8]^表明脂肪酸氧化代谢的关键代谢物肉碱、三羧酸循环的关键中间体如马来酸、富马酸、α-酮戊二酸的含量均升高，提示肿瘤细胞能量代谢需求进一步提高，三羧酸循环流量剧增。

### 1.2 氨基酸代谢的异常

氨基酸代谢重编程在肿瘤的增殖、侵袭、耐药和免疫调节中发挥关键作用。代谢组学研究发现，谷氨酰胺-谷氨酸代谢在肺癌细胞中高度活跃^[6]^，可为三羧酸循环提供碳源，并通过关键酶谷氨酸脱氢酶1（glutamate dehydrogenase 1, GLUD1）调控肿瘤细胞的获得性耐药性^[11]^。此外，有研究者^[7,12]^发现肺癌组织的半胱氨酸和甘氨酸水平升高，促进谷胱甘肽（glutathione, GSH）合成，提高肿瘤细胞的抗氧化能力，使其能够抵抗氧化应激和化疗诱导的细胞死亡。不仅如此，一项针对肺腺癌的机制研究^[13]^也揭示了致癌Kirsten大鼠肉瘤病毒癌基因同源物（Kirsten rat sarcoma viral oncogene homolog, *KRAS*）突变的代谢脆弱性，通过代谢组学方法，研究者们发现突变的*KRAS*显著增加了肺腺癌细胞内的胱氨酸和GSH水平，负责特异性摄取胱氨酸的转运蛋白溶质载体家族7成员11（solute carrier family 7 member 11, SLC7A11）的过度表达是其主要原因。代谢组学联合蛋白质组学的证据^[14]^也提示，相比于正常肺组织，肺腺癌组织中与GSH生物合成、GSH循环、氮平衡相关的酶出现显著上调。同时，精氨酸、鸟氨酸和多胺代谢产物（如腐胺、亚精胺、精胺）的上调，有助于维持肿瘤细胞的快速增殖和侵袭能力，而脯氨酸的增加可能促进基质重塑和肿瘤微环境的适应^[6,15,16]^。另一方面，肺癌组织中抗氧化相关氨基酸（如半胱氨酸、甲硫氨酸亚砜）和一碳代谢相关氨基酸（如丝氨酸、甘氨酸）的异常，可能影响肺癌细胞对活性氧（reactive oxygen species, ROS）的应激反应和DNA甲基化状态^[17]^。基于这些代谢特征，谷氨酰胺/谷氨酸比值、多胺代谢产物、SLC7A11-GSH轴等可作为肺癌的潜在生物标志物，用于早期诊断和预后评估。此外，CB-839^[18]^（谷氨酰胺酶抑制剂）、GLUD1抑制剂^[11]^、SLC7A11抑制剂^[13]^等靶向代谢治疗策略已在体内体外实验模型中展现出良好前景，结合代谢组学与多组学数据，有望进一步提高肺癌精准诊疗的效果。

### 1.3 脂质代谢重编程

脂质代谢的重编程在维持肺癌细胞膜稳定性、信号传导、能量供给及抗氧化防御中发挥关键作用。研究^[7,19]^表明，肺癌组织中磷脂酰胆碱和甘油磷脂水平显著升高，提示细胞膜合成及流动性增强，有助于肿瘤的侵袭和转移。此外，胆固醇合成及酯化代谢的上调可促进细胞膜结构的重塑，并增强肿瘤细胞对氧化应激的耐受能力，而花生四烯酸和二高亚麻酸的积累则与炎症反应及促肿瘤信号通路的激活密切相关^[6,15]^。另一方面，有研究报道^[15]^显示肉碱和柠檬酸含量降低，提示脂肪酸β-氧化（fatty acid oxidation, FAO）受损，使肿瘤细胞更依赖糖代谢作为主要能量来源。脂质过氧化的异常调控也是肺癌细胞代谢重编程的重要一环，Godzien等^[20]^通过代谢组学发现肺癌细胞中长链和短链的氧化磷脂酰胆碱（oxidized phosphocholine, oxPC）含量均显著升高，提示某些肺癌细胞抗氧化能力的降低。基于脂质代谢的这些变化，靶向胆固醇酯化、FAO通路及脂质过氧化或许可以成为新的治疗策略，其中硬脂酰辅酶A去饱和酶1（stearoyl-CoA desaturase 1, SCD1）抑制剂^[21]^、长链脂酰辅酶A合成酶4（long-chain acyl-CoA synthetase 4, ACSL4）调控剂^[22]^等靶向脂质代谢的药物均在肿瘤治疗研究中展现出潜力，结合代谢组学分析，有望进一步优化肺癌的精准治疗方案。

### 1.4 核苷酸代谢改变

肺癌细胞的核苷酸代谢重编程在维持其快速增殖、DNA修复及应对代谢压力方面起重要作用。代谢组学研究^[6,7,15]^发现，肺癌组织中嘌呤和嘧啶合成通路高度激活，导致腺苷、肌苷、黄嘌呤、鸟苷等核苷代谢物显著上调，这些变化为肿瘤细胞提供充足的核苷酸以支持DNA和RNA的合成。此外，戊糖磷酸途径（pentose phosphate pathway, PPP）的增强促进了核糖-5-磷酸（ribose-5-phosphate）的积累，为核苷酸合成提供前体，同时增加NADPH的生成，增强细胞的抗氧化能力^[8]^。另一方面，尿苷和胞苷水平升高，提示嘧啶代谢的加速，而嘌呤降解产物（如尿酸）在部分肺癌组织中增加，表明核苷酸循环速率加快^[23,24]^。有研究^[25]^发现，肺癌细胞对核苷酸拮抗剂类的化疗药物（如5-氟尿嘧啶和甲氨蝶呤）的敏感性，与核苷酸合成酶的表达水平密切相关，提示靶向核苷酸代谢可能是克服肺癌耐药性的有效策略。此外，CD73介导的腺苷信号通路在肺癌微环境中上调，随后的实验^[26]^证明腺苷的积累可抑制T细胞活性，促进免疫逃逸，表明阻断CD73-腺苷轴可能增强免疫治疗效果。综合来看，肺癌的核苷酸代谢重编程不仅为细胞快速增殖提供物质基础，还通过调节TIME和抗氧化应激，影响治疗敏感性，提示其在精准医疗中的重要作用。

### 1.5 代谢重编程与免疫微环境的交互作用

近年来，越来越多研究揭示肺癌细胞代谢重编程与其TIME之间存在复杂的相互作用。肿瘤细胞通过重塑葡萄糖、脂质和氨基酸代谢路径，不仅满足其快速增殖的需求，还可以调节肿瘤微环境中免疫细胞的功能^[27]^。例如，乳酸的过度产生可导致肺癌组织局部微环境的pH下降，从而抑制CD8^+^ T细胞和自然杀伤（nature killer, NK）细胞的杀伤活性，同时促进调节性T细胞（regulatory T lymphocytes, Tregs）和M2型巨噬细胞的免疫抑制表型^[10]^。除此之外，肺癌细胞向TIME中排放的另一种小分子代谢物琥珀酸，可激活巨噬细胞的琥珀酸受体（succinate receptor 1, SUCNR1），活化PI3K-HIF-1α轴，从而促进其向抑制免疫的M2表型极化^[28]^。这些机制共同构成了代谢与免疫互作的调控网络，深刻影响肺癌的免疫逃逸与免疫治疗响应。

### 1.6 空间代谢组学在肺癌异质性研究中的应用

质谱成像（mass spectrometry imaging, MSI）是肿瘤代谢组学中的一项新兴技术。解吸电喷雾电离（desorption electrospray ionization, DESI）和基质辅助激光解吸电离（matrix-assisted laser desorption ionization, MALDI）是MSI常用的2项技术，能以接近单细胞分辨率的方式识别肿瘤细胞中的数百种小分子代谢物，从而能够专注于肿瘤异质性以及肿瘤微环境中不同细胞之间的信号通信^[29]^。Cai等^[30]^指出，基于空间分布的代谢组学分析能够揭示NSCLC不同亚型间的潜在代谢脆弱性。最近的一项泛癌种研究^[31]^开创了一种新的单细胞空间代谢组学方法，成功实现了对包括A549肺癌细胞在内的4种细胞系进行多种小分子代谢物的空间原位成像，揭示了不同癌细胞之间的显著空间代谢差异以及与成纤维细胞共培养前后的代谢差异。另一项针对NSCLC不同亚型的研究^[32]^则通过将MALDI-MSI技术与组织病理图谱结合，构建了组织结构指导下的空间代谢分析策略，实现了对NSCLC亚型的精细分型，此研究发现，腺癌与鳞癌在脂质代谢通路上的空间表达存在显著差异，部分关键代谢物如磷脂类物质呈现出区域特异性的高表达。此外，一项针对肺鳞癌的研究^[33]^通过空间代谢数据与临床生存资料的交互分析，发现代谢异质性与肿瘤浸润性T淋巴细胞（tumor infiltrating lymphocytes, TILs）密切相关，代谢异质性越高，TILs水平越低、生存越差。

总而言之，空间代谢图谱能够直观反映肿瘤内部复杂的代谢重编程模式，为理解肺癌进展、耐药和免疫逃逸机制提供了新的研究维度。

## 2 代谢组学在肺癌早期诊断中的应用

肺癌的早期诊断对于提高生存率至关重要。然而，现有的LDCT筛查存在假阳性率高和过度诊断的问题^[34]^。代谢组学通过分析肺癌患者和健康对照的血清、尿液、呼气冷凝液（exhaled breath condensate, EBC）、组织及支气管肺泡灌洗液（bronchoalveolar lavage fluid, BALF）等样本中的代谢物变化，为肺癌的早期检测提供了新的方向。

血清/血浆代谢组学具有非侵入性、易采集的特点，适用于动态监测，但受饮食、生活方式和个体差异影响较大。多项研究^[35-37]^发现，甘油磷脂、胆固醇衍生物、色氨酸代谢物、5′-磷酸吡哆胺、油酸等可作为肺癌相关的代谢标志物，反映机体循环代谢紊乱以及肿瘤微环境的代谢异常。此外，一项多组学研究^[38]^发现，NSCLC患者血浆代谢物花生四烯酸和13-羟基十八碳二烯酸（13-S-hydroxyoctadecadienoic, 13-HODE）可作为生物标志物，偏最小二乘法判别分析（partial least squares discrimination analysis, PLS-DA）方法的曲线下面积（area under the curve, AUC）可达到0.91，且与Akt通路显著相关。另一项代谢组学联合转录组学的研究^[39]^发现6种代谢物作为生物标志物组合（次黄嘌呤、肌苷、L-色氨酸、吲哚丙烯酸、酰基肉碱C10:1和lysoPC18:2），其AUC可达0.99，敏感度和特异度均超过0.98；通过对代谢组学数据与转录组学发现的2000多种差异基因交互进行分析，研究者们成功构建出NSCLC相关的代谢路径-基因调控网络。

尿液代谢组学有无创、代谢物浓度高的特点，适合大规模筛查，但其可能受到肾功能、液体摄入量、药物影响等因素干扰，需要设法克服这些问题才可能应用于肺癌筛查。例如，有研究^[40]^发现5-甲基-2-呋喃酸在非吸烟女性肺癌患者尿液中显著升高，可作为潜在生物标志物，一碳代谢和氧化应激途径等的系统性变化也可能与肺癌风险相关。在另外一项泛癌种的尿液代谢组学研究^[41]^中，肺癌和非肺癌被算法最小绝对收缩和选择算子（least absolute shrinkage and selection operator, *LASSO*）挑选出的18种代谢物较好地区分，其验证集的AUC值达到0.88。

呼气冷凝液及其中的挥发性有机化合物（volatile organic compounds, VOCs）的代谢组学分析，有望成为一种具有前景的无创肺癌早期诊断技术。该方法基于机体代谢活动产生的VOCs在肺部交换后被呼气携带的原理，能够反映肺癌患者代谢紊乱及细胞应激状态的变化。不同病理状态下的人体呼气中会产生特异性的VOCs图谱，在肺癌患者中也表现出不同于健康人群的代谢特征^[42]^。Wang等^[43]^的研究揭示，肺癌患者（*n*=24）对比健康对照（*n*=26），其EBC中上调的代谢物包括氨基酸及其衍生物（如苯丙氨酸和色氨酸等）和脂肪酸类（如十三烯酸、十六碳二烯酸等），下调的代谢物主要有3,4-亚甲基苯甲酸、2-异丙基苹果酸等，基于差异分析结果开发的预测模型，其灵敏度达到86.2%，特异性为83.3%，准确率为84.9%，应用前景广阔。另外，还有研究者利用基于NMR的代谢组学平台研究了手术前后NSCLC患者的EBC和痰液代谢物改变，发现术后单磷酸腺苷和N1, N12-diacetylspermine出现明显上调^[44]^；此外，还有研究^[45]^聚焦于NSCLC患者放疗后出现肺损伤的不良反应，发现肺损伤患者的EBC表现出与三羧酸循环相关的脂质、氨基酸和糖类能量代谢的明显改变，提示它们可能作为预测放疗相关肺损伤的标志物。后两项研究可能为进一步探索肺癌早期筛查及预测治疗反应的非侵入性工具提供坚实基础。

组织样本直接来源于肿瘤组织和正常组织，特异性高，适用于肿瘤分型、分期及预后评估，但作为临床诊断的金标准，由于获取困难，需要进行有创操作，不适合作为早期筛查手段。且肿瘤组织存在空间异质性，不同部位的代谢状态可能存在差异。

BALF的代谢组学因BALF样本直接来源于肺部，可提供最接近肿瘤微环境的代谢信息，适用于检测微小病变。一项研究^[46]^显示，在肺癌患者BALF代谢物中丝氨酸和丁二酰基肉碱（succinylcarnitine, C4DC）显著变化，丝氨酸和C4DC在预测模型中的AUC分别为0.843和0.785，提示其与肿瘤细胞的生长需求和线粒体代谢重编程相关。此外，另一项研究^[47]^收集了24例肺癌和30例非肺癌患者的BALF样本（支气管镜技术具有侵入性，因此无法获得健康对照样本），发现甘油和磷酸在肺癌BALF中显著下调，它们的AUC均大于0.75，具有早期诊断标志物的潜力。

不同生物样本各具优势，但也存在各自的局限性，具体内容已经总结在表1^[48-52]^中。这些生物样本的代谢组学数据相结合，有望提高肺癌早期诊断的准确性，为精准医疗提供新策略。

**表1:** 不同样本类型在基于代谢组学的肺癌早诊中的比较

Sample type	Advantages	Limitations	Applications	Reference
Serum/Plasma	Easy collection, well-standardized; rich in metabolites; suitable for large-scale studies	Strongly affected by diet and medications; low specificity for lung lesions	Candidate biomarker screening; integration with clinical indicators	Liang et al., 2024, Int J Med Sci^[48]^
Urine	Non-invasive, high patient compliance; stable metabolite composition; reflects systemic metabolism	High inter-individual variability; influenced by renal function; lower specificity	Pre-screening or auxiliary testing to improve sensitivity	Bax et al., 2019, Cancers (Basel)^[49]^
EBC	Non-invasive; reflects local lung metabolism; suitable for VOC and dynamic monitoring	Extremely low metabolite concentrations; poor reproducibility; technical standardization lacking	Dynamic monitoring of pulmonary metabolism; aid for early detection	Binson et al., 2025, Med Gas Res^[50]^
Tumor tissue	High tissue specificity; directly reflects tumor microenvironment metabolism	Requires invasive sampling; not suitable for screening; strict preservation conditions	Mechanistic studies; biomarker discovery; molecular subtyping	Zang et al., 2022, J Proteome Res^[51]^
BALF	Derived directly from lung lesions; high specificity for local inflammation and tumor status	Invasive collection; technically complex; limited sample volume; unsuitable for screening	Characterization of local metabolic changes; mechanistic exploration or preoperative assessment	Kalkanis et al., 2022, Diagnostics (Basel)^[52]^

EBC: exhaled breath condensate; BALF: bronchoalveolar lavage fluid; VOC: volatile organic compounds.

## 3 代谢组学在研究肺癌耐药机制中的应用

部分患者在接受靶向治疗或化疗的过程中，其肺癌细胞会通过代谢重编程适应治疗压力，从而发展出耐药性。近年来，代谢组学的应用为解析耐药机制提供了新的研究工具，并揭示了多个关键的代谢适应机制。针对这些代谢变化的靶向干预，可能为克服耐药性提供新的治疗策略。

### 3.1 氧化还原代谢的适应性重编程与耐药性

肺癌细胞在耐药过程中会调整氧化还原代谢，以降低ROS水平，避免化疗或靶向治疗诱导的细胞凋亡。代谢组学研究^[53]^表明，在顺铂（Cisplatin）耐药的肺癌脑转移细胞中，GSH过氧化酶4（glutathione peroxidase 4, GPX4）介导的GSH高消耗可减少脂质过氧化，从而抑制铁死亡，增强耐药性。此外，多西他赛（Docetaxel）耐药细胞表现出半胱氨酸摄取减少，导致GSH水平下降，进一步增强耐药性。实验发现，补充半胱氨酸可恢复GSH水平，重新平衡细胞的氧化还原状态，并逆转耐药表型^[12]^。这些研究表明，调控GPX4活性或补充抗氧化代谢物，可作为克服化疗耐药的重要策略。另外，氧化磷酸化也是调控肺癌细胞耐药性的一大途径。研究^[54]^发现，核受体共激活因子4（nuclear receptor coactivator 4, NCOA4）介导的铁蛋白自噬（ferritinophagy）促进了铁-硫簇蛋白的合成，进而增强电子传递链活性，使耐药细胞的氧化磷酸化功能更强。

### 3.2 糖代谢重编程与耐药性

糖代谢重编程是肿瘤细胞适应生存压力的重要机制，在肺癌耐药过程中尤为显著。Warburg效应使肿瘤细胞主要依赖无氧糖酵解供能，即便在氧气充足的条件下仍偏向乳酸发酵。Hayes等^[10]^利用代谢组学等一系列手段发现，以无氧糖酵解为主的代谢方式不仅为肿瘤细胞提供三磷酸腺苷（adenosine triphosphate, ATP）和生物合成所需的中间代谢物，还通过乳酸的积累塑造免疫抑制性微环境，削弱T细胞和NK细胞的抗肿瘤功能，进而促进免疫逃逸和耐药性的形成。还有研究^[55]^表明乳酸还能诱导M2型巨噬细胞极化，增强免疫抑制特性，从而降低肺癌细胞对化疗和靶向治疗的敏感性。除此之外，在表皮生长因子受体-酪氨酸激酶抑制剂（epidermal growth factor receptor-tyrosine kinase inhibitors, EGFR-TKIs）耐药的NSCLC细胞中，葡萄糖转运蛋白1（glucose transporter type 1, GLUT1）和己糖激酶2（hexokinase 2, HK2）表达上调，促进葡萄糖摄取和糖酵解速率的增加，同时乳酸外排依赖的单羧酸转运蛋白4（monocarboxylate transporter 4, MCT4）亦显著升高，维持耐药细胞在高乳酸环境中的存活^[56]^。此外，糖酵解增强可激活PI3K/Akt/mTOR通路，通过促进抗凋亡蛋白，如B淋巴细胞瘤2家族蛋白（B cell lymphoma-2, BCL-2）的表达来增强耐药性，同时抑制线粒体依赖的细胞凋亡通路，降低细胞对药物的敏感性^[57]^。因此，靶向糖酵解或乳酸转运的关键蛋白，有望逆转肺癌细胞耐药性，并增强化疗和靶向治疗的疗效。

### 3.3 氨基酸代谢重编程与耐药性

EGFR-TKIs是*EGFR*突变NSCLC的标准治疗方案，但耐药性严重限制了其疗效。代谢组学分析^[11]^发现，EGFR-TKIs耐药细胞和多西他赛耐药细胞高度依赖谷氨酰胺代谢，GLUD1在耐药细胞中上调，增强了谷氨酰胺向α-酮戊二酸转化，并促进氧化磷酸化，赋予耐药细胞更强的能量适应能力。除了谷氨酰胺代谢，乙酰胆碱信号通路也在EGFR-TKIs耐药过程中发挥作用。Nie等^[58]^的研究表明，在EGFR-TKIs治疗下胆碱乙酰转移酶（choline acetyltransferase, ChAT）表达增加，导致乙酰胆碱积累，并通过激活M3型毒蕈碱受体促进耐药细胞存活。这些研究表明，代谢组学可用于识别EGFR-TKIs耐药相关的代谢适应机制，并提供新的干预策略。

### 3.4 脂质代谢重塑在耐药中的作用

脂质代谢不仅影响肿瘤细胞的膜组成和信号传导，还在耐药性形成中起关键作用。先前的一项研究^[59]^发现NSCLC细胞中，脂肪酸合成酶（fatty acid synthetase, FASN）呈高水平表达，催化生成的16碳饱和脂肪酸即棕榈酸，可通过促进EGFR的棕榈酰化修饰，从而激活其下游信号传导通路，参与诱导TKIs相关的获得性耐药机制。此外，在肉碱棕榈酰转移酶1A（carnitine palmitoyltransferase 1A, CPT1A）介导的FAO通路过度激活时，肺癌细胞可抑制铁死亡，并通过NRF2/GPX4轴增强抗氧化能力，进而提高对靶向治疗的耐受性^[60]^。此外，Zhang等^[61]^在*Advanced Science*上发现，EGFR-TKIs治疗后NSCLC细胞可通过上调二肽基肽酶4（dipeptidyl peptidase 4, DPP4），进而促进CPT1A表达，促进脂肪酸摄取和氧化，从而形成“持存细胞”，进一步提示DPP4-CPT1A-FAO是重要的耐药生存路径。

### 3.5 耐药机制的网络效应

多组学研究^[62-64]^持续揭示NSCLC的获得性耐药可能不是由某一条代谢通路主导，而是由多个代谢路径间复杂的网络重塑共同驱动的“网络效应”。在EGFR-TKIs耐药机制中，多项研究^[11,65]^显示，耐药细胞对谷氨酰胺的依赖显著增强，通过上调GLUD1促进其向α-酮戊二酸转化，从而强化三羧酸循环和氧化磷酸化，满足高能量需求；同一时间，GLUT1与HK2的上调又增强了葡萄糖摄取与糖酵解流通速率，使糖代谢重编程产物乳酸在微环境中堆积；乳酸积聚不仅为肺癌细胞提供持续代谢优势，还能通过调节TIME中的T细胞与巨噬细胞功能，进一步促进免疫逃逸与耐药性形成。

值得注意的是，脂质代谢通路与氨基酸代谢通路之间存在显著的交叉调控。GPX4介导的脂质过氧化抑制作用依赖于GSH的持续供给，而GSH的合成则依赖于谷氨酸与半胱氨酸的代谢。这一代谢交互不仅维持细胞的氧化还原稳态，还通过抑制铁死亡提高耐药细胞的生存适应性。此外，FAO通路中DPP4-CPT1A的激活也通过增强NRF2-GPX4轴的活性来促进ROS清除和抗氧化防御，进一步增强对药物诱导的细胞死亡的抵抗。

这些复杂的代谢重编程现象往往难以通过单组学手段完整阐释。整合代谢组学、蛋白质组学及转录组学等多组学数据，有助于系统识别关键代谢通路的上下游调控因子及其协同关系。蛋白质组层面可揭示代谢酶表达水平的变化，代谢组学可精确解析其代谢产物积累模式，两者结合可精确锁定潜在的耐药节点。因此，基于多组学的“网络型”耐药机制研究，正逐步成为肺癌耐药机制解析和治疗靶点发现的重要方向。

## 4 肺癌代谢组学临床应用的挑战与对策

肺癌代谢组学的临床转化面临多重挑战。首先，代谢组学分析的步骤和环节繁多，从样本的采集、运输、储存、预处理，到分析平台的选择、仪器参数的设置，再到数据的采集、处理和统计分析方法，各个环节都缺乏统一的标准化操作规范^[66]^，这是制约代谢组学临床应用的首要瓶颈。同时，目前大多数研究，特别是生物标志物发现阶段的研究，样本量相对较小，且多为回顾性或病例-对照设计，缺乏大规模前瞻性验证以确认临床效能。此外，已发现的代谢标志物常缺乏肺癌特异性，可能出现于其他疾病状态^[48]^，并易受个体生理、生活方式（如吸烟）等混杂因素干扰，增加了标志物筛选难度^[67-69]^。许多差异代谢物的生物学机制阐释亦不充分，限制了临床意义的准确评估，加之现有分析平台的成本效益与临床可操作性也制约其广泛应用。为应对这些挑战，亟需推动行业标准化共识与指南的制定并加强多中心合作，加大对大规模前瞻性临床验证的投入并建立标准化生物样本库。同时，应通过更全面的对照研究和机制探索提升标志物特异性，在研究设计与数据分析中严格控制与校正混杂因素，并鼓励代谢组学与功能实验紧密结合以深入阐明分子机制，积极研发成本更低、操作更便捷的检测技术，全面评估其临床增量价值与经济学效益。

综上所述，代谢组学在肺癌研究中发挥着越来越重要的作用。通过对糖代谢、氨基酸代谢、脂质代谢和核酸代谢等多个层面的全面解析，代谢组学能够深入揭示肺癌的早期诊断、疾病发展及耐药机制。其独特的优势在于能够反映肿瘤细胞的代谢特征和动态变化，为肺癌的早期筛查、预后评估以及耐药性克服提供了新的研究方向。未来，随着机制研究的深入以及代谢靶向药物的开发，代谢组学将在肺癌的精准治疗中发挥至关重要的作用，为克服肺癌治疗中的难题提供创新性的解决方案。
